# Sentiment analysis of epidemiological surveillance reports on COVID-19 in Greece using machine learning models

**DOI:** 10.3389/fpubh.2023.1191730

**Published:** 2023-07-18

**Authors:** Christos Stefanis, Elpida Giorgi, Konstantinos Kalentzis, Athanasios Tselemponis, Evangelia Nena, Christina Tsigalou, Christos Kontogiorgis, Yiannis Kourkoutas, Ekaterini Chatzak, Ioannis Dokas, Theodoros Constantinidis, Eugenia Bezirtzoglou

**Affiliations:** ^1^Laboratory of Hygiene and Environmental Protection, Department of Medicine, Democritus University of Thrace, Alexandroupolis, Greece; ^2^Pre-Clinical Education, Laboratory of Social Medicine, Medical School, Democritus University of Thrace, Alexandroupolis, Greece; ^3^Laboratory of Microbiology, Medical School, Democritus University of Thrace, Alexandroupolis, Greece; ^4^Laboratory of Applied Microbiology, Department of Molecular Biology and Genetics, Democritus University of Thrace, Alexandroupolis, Greece; ^5^Laboratory of Pharmacology, Medical School, Democritus University of Thrace, Alexandroupolis, Greece; ^6^Department of Civil Engineering, Democritus University of Thrace, Komotini, Greece

**Keywords:** sentiment, classification, COVID-19, Facebook, public health, machine learning, natural language processing

## Abstract

The present research deals with sentiment analysis performed with Microsoft Azure Machine Learning Studio to classify Facebook posts on the Greek National Public Health Organization (EODY) from November 2021 to January 2022 during the pandemic. Positive, negative and neutral sentiments were included after processing 300 reviews. This approach involved analyzing the words appearing in the comments and exploring the sentiments related to daily surveillance reports of COVID-19 published on the EODY Facebook page. Moreover, machine learning algorithms were implemented to predict the classification of sentiments. This research assesses the efficiency of a few popular machine learning models, which is one of the initial efforts in Greece in this domain. People have negative sentiments toward COVID surveillance reports. Words with the highest frequency of occurrence include government, vaccinated people, unvaccinated, telephone communication, health measures, virus, COVID-19 rapid/molecular tests, and of course, COVID-19. The experimental results disclose additionally that two classifiers, namely two class Neural Network and two class Bayes Point Machine, achieved high sentiment analysis accuracy and F1 score, particularly 87% and over 35%. A significant limitation of this study may be the need for more comparison with other research attempts that identified the sentiments of the EODY surveillance reports of COVID in Greece. Machine learning models can provide critical information combating public health hazards and enrich communication strategies and proactive actions in public health issues and opinion management during the COVID-19 pandemic.

## 1. Introduction

The pandemic crisis that broke out due to the COVID-19 disease not only tested the limits of the health systems in all countries but also other aspects of the citizens' political, economic and social life. Dissemination of information about the hazard of COVID-19, infections, deaths and the respective measures and actions toward mitigating consequences by each country has been considered of great importance (1, 2).

The enormous amount of data produced in the era of COVID has manifested a new research path that is; the intensification of Big Data analytics and Artificial Intelligence toolbox, mainly the implementation of machine learning algorithms and predictive modes. The goal is to apply models in order to predict the risk, to forecast COVID pandemic waves, to diagnose results, and to detect information and misinformation activities related to public health management in Social Networking Sites (SNS), as well the society (3).

One of the main communication channels of public authorities during a crisis, like a health crisis, is social media. Especially since, in today's era, the internet offers speed and immediacy in disseminating critical information from the state to the citizens. Planning and drawing up a communication strategy through social media is critical. It requires a high degree of expertise to not produce opposite results, such as misinformation and confusion among citizens. Therefore, social media became a powerful weapon in state and health agencies' arsenal against the pandemic crisis, intending to inform citizens at individual and state levels about the evolution of the pandemic, individual protection measures, restrictive measures and upcoming health policies which were to be followed (4, 5).

In addition, issues and policies related to the evaluation of health services, the dissemination of preventive health, psychological and educational actions against COVID-19 were disseminated to the public and extensively commented on through Social Networking Sites. The next step in utilizing these online SNS is knowledge as well as data mining for early warning and detection of COVID-19 incidents, the emotional orientation of the public regarding the whole range of actions during the pandemic crisis, and filtering of information and misinformation from the public (6, 7).

Sentiment analysis can decipher people's opinions, emotions and sentiments. Political sciences, marketing and sales use sentiment analysis to improve products and services following up customer reviews. In the medical field, sentiment analysis is used to spot and extract opinions and sentiments on social media on issues regarding mental health, epidemiology, new medical treatments, drugs and supplements, patient forums, and pharmacovigilance (8).

The use of social media should also be discussed since sentiment orientation analysis utilizes such data originating from social discussions. However, why is social media used in public health matters? The answer is conclusively given by the fact that critical information can be extracted from these, highlighting the population's demographic, spatial and socio-economic disparities. However, using these data and big data for infectious disease surveillance should be evaluated for their reliability and credibility in research initiatives concerning public health policy, public opinion and trends, health crisis management, pandemics and infectious disease control and surveillance (9, 10).

Health issues surveillance is an additional role of SNS in their usefulness as a knowledge tool in public health. Moreover, analyzing features and generating real time data of pharmacovigilance, information or misinformation surveillance, and tracking health behavior issues like parents' health literacy skills are also embodied in sentiment analysis in the public health domain (11–13).

In the middle of the last decade, the Centers for Disease Control and Prevention (CDC) has recognized the role of health-related data that signal a possibility of an outbreak or the initial development of an epidemic outbreak and can signify a disturbance in public health (syndromic surveillance^1^). The speed of exploiting this data with the right tools, such as sentiment analysis, due to a 1–2 weeks lag between diagnosis and when this information becomes part of published statistics, has resulted in a practical approach to epidemic detection. Subsequently, the formation or combination of surveillance systems aimed at detecting a particular disease and prompting health officials to take appropriate measures and communicate health policies to prevent outbreaks can be realized (14).

National Public Health Organization (EODY) in Greece supervise all the public health services related to communicable and chronic diseases and implement all the necessary actions for epidemiological surveillance, risk assessment, scientific consultation, dissemination and communication strategies to inform the population about health issues, like COVID-19 surveillance reports and data (15). The main innovations of our research include the following:

- Analyze the sentiment orientation of Greek citizens' comments regarding the surveillance reports of COVID-19 during the last winter of the pandemic (December 2021-January 2022).
- Determine various issues and topics the public discussed on EODY Facebook page.
- Apply machine learning algorithms to predict the sentiment orientation of public opinion toward an even more concise picture of the given period of time.
- Study people's emotions in Facebook posts to recognize and interpret how classification models can support the understanding of public perception toward the COVID-19 pandemic.

The current contribution of this research approach is to develop and propose a basis for identifying issues about the impact of the surveillance reports of COVID. Extracting public sentiments from Facebook provides the raw material to decision makers in Greek public health agencies for compassing new communication strategies based on digital communications via social media and public reaction monitoring.

### 1.1. Related work

In our effort to identify and highlight the applications of sentiment analysis in the field of public health and the management of infectious diseases, pandemics and the combination of such tools in existing public health management and surveillance systems, we conducted a search on Scopus for research papers. The aim is 2-fold: to identify works in the above field, namely the combination of sentiment analysis with related initiatives in public health. Additionally, we realize science mapping to reveal the research content of such approaches by running a co-occurrence analysis, a bibliometric tool and spotlighting the linkage of sentiment analysis and its use in public health (Supplementary Figure 1).

**Figure 1:**
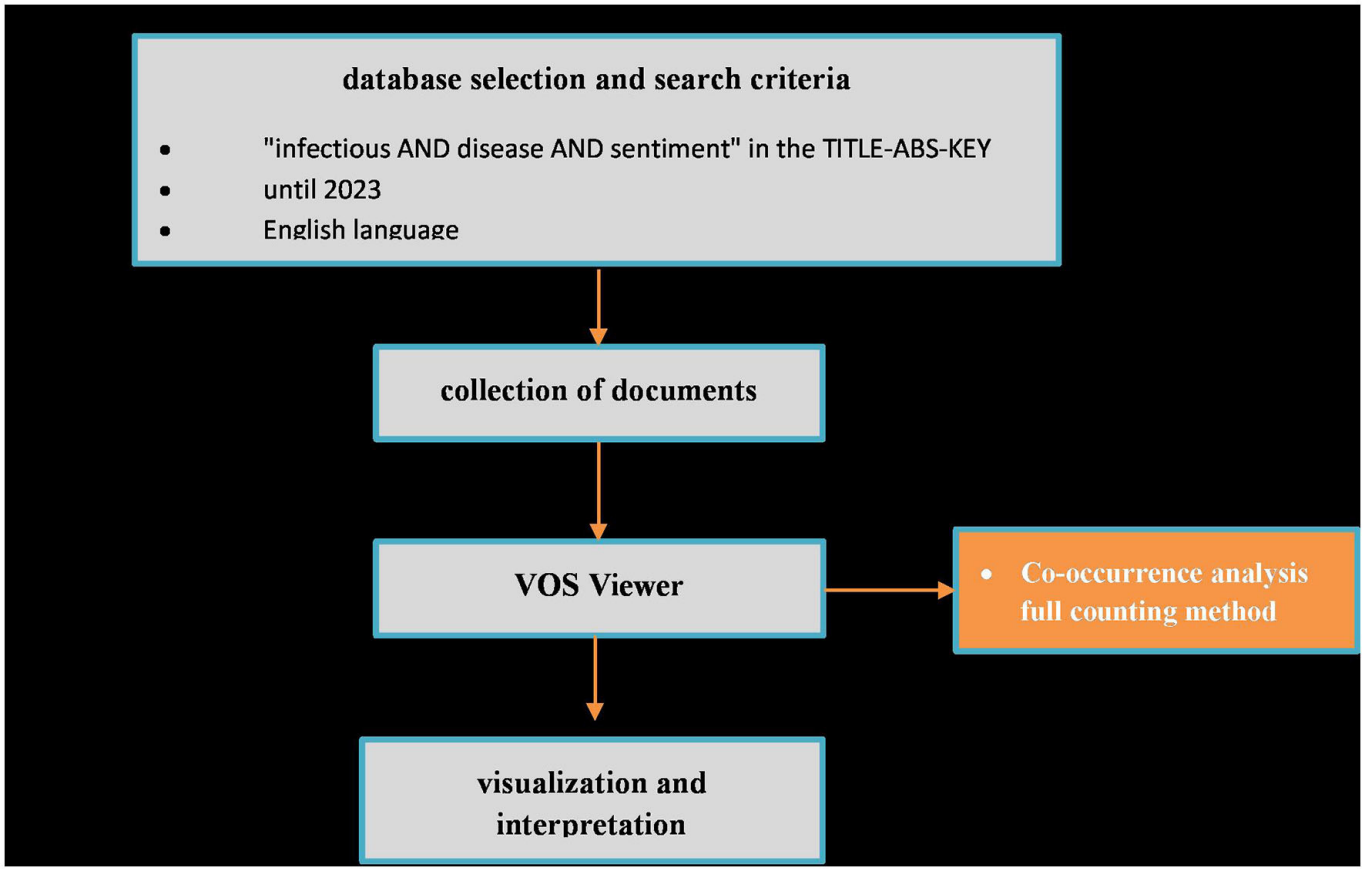
Research workflow diagram of bibliometric analysis.

The phrase “infectious AND disease AND sentiment” in the TITLE-ABS-KEY search field was used for scanning the Scopus database and the bibliometric approach. The year 2023 was excluded, while only English manuscripts and various document types were included. The VOS Viewer software was applied to visualize the results and create a bibliographic map^2^ (Figure 1). The full counting method was further used in the co-occurrence analysis of the keywords in the title, abstract, and text of the manuscripts. Briefly, 1,380 keywords were initially extracted, but setting the minimum number of occurrences to 5, 66 were included. The bibliographic network revealed 4 clusters with 54 keywords on the map (Supplementary Figure 1) (16, 17). Keywords, clusters, weight score and number of occurrences of each item in the bibliographic map are listed in Supplementary Table 2.

As stated above, the bibliographic map categorizes terms into four significant clusters with respective colors. The color of each word is dictated by the cluster to which it belongs. Moreover, the closer two terms are spotted on the map, the stronger their relatedness. The yellow cluster contains words referring to the COVID-19 pandemic crisis, such as COVID-19, pandemic, coronavirus and coronavirus infection. The green cluster includes terms related to vaccines and vaccination. The blue cluster circulates items like infectious disease, disease surveillance, communicable disease and disease transmission. At the same time, the red one encompasses terms like sentiment analysis, infectious disease, data mining, and social media platforms.

Interpreting the bibliographic network and the included keywords, one should note the engagement and the boost that the COVID period offered in the surveillance, management and monitoring of infectious diseases via social media and online media platforms with computational linguistic applications like sentiment analysis. Such applications were already in use; however, the pandemic period spotlighted extra interest and research value. Public perception, opinions, patients' experience, opinion mining and sentiment analysis are established as vital tools in infoepidemics, infodemiology, and infoveillance's arsenal (18–22).

In the current literature regarding sentiment analysis studies concerning public health, COVID-19 and data generated and circulated in social media focused on data extracted from Twitter. A proposed methodological framework addressed the problem of public fear during the COVID-19 peak in the United States of America. The characteristics of this framework are based on machine learning algorithms, namely logistic regression and Naïve Bayes, as well as text analysis methods (23).

In the past several years, COVID-19 has revolutionalized many aspects of everyday life. One aspect is the working environment, especially in developed countries, where remote work has become common practice. This transition to new labor standards at the commencement of the world epidemic using sentiment analysis was the goal of another contribution (24).

Public sentiments followed the deviation of the three pandemic waves in Croatia, a fact revealed in a recent study. This study underlined the significant impact of COVID-19 on the psychological side effects of society. Twitter data, sentiment analysis, and machine learning algorithms were implemented to detect, among others, the polarity of Croatian public opinion (25).

A research study aimed at analyzing Twitter data on citizens' attitudes toward vaccination policies and issues in the USA. The findings revealed positive attitudes toward vaccination and necessary safety measures against COVID-19 (26). Furthermore, the justification of sentiment analysis using data from SNS is also manifested in another proposed research. The outcome emphasized that emotional state and sentiment polarity information during the pandemic can support communication strategies and public guidance (27).

Further research focused on Twitter data, COVID-19 pandemic and governmental management actions by studying public sentiment orientation and emotional positions (28). Besides the sentiment analysis, this research team developed a decision support system to scrutinize Facebook posts to aid the decision making of public health agencies during the diverse health crisis of COVID-19.

In recent years, the sentiment analysis emerging from text mining of various Social Networking Sites has been increasing in Greek scientific literature in multiple research fields like informatics and management, business administration, political sciences, computer engineering and statistics (29–35). In the field of public health, there are very few research efforts and even fewer studies focusing on COVID-19 and Facebook comments (7, 36). Considering the data sources, social media platforms, disease-specific communities, patient forums, blogs, and electronic health records and platforms are the most common. Among the most common platforms for the exploitation of health and patient experience data is Twitter (37, 38). The primary usage of the Twitter platform in health-related issues like public health and infectious diseases is for content analysis and secondary for surveillance (39). The prospective and promising usage of the Internet data sources in infectious disease and epidemic surveillance is realized increasingly as an effective tool. Data's spatial and temporal distribution can be utilized for internet-based surveillance, disease forecasting and modeling (40).

In the healthcare industry, opinion mining and sentiment extraction are vital. Patients express their opinions and sentiments regarding old and contemporary treatments, medicines and public health services. In this vein, a research study highlights the analysis of tweets concerning diabetes. Additionally, a health web forum was utilized to extract the sentiments of people and the differentiation of genders diagnosed with HIV and the respective issues confronting their lives (41, 42).

## 2. Materials and methods

### 2.1. Overall methodological approach

The analysis of sentiment orientation includes a sequence of steps to ensure the correct depiction of the properties evaluated based on the categorization of emotions. Briefly, collecting data from the source, which in this particular study is Facebook, is the first step. Next is data preprocessing, which usually refers to removing words, grammar, and syntactical phrases to clean up a sentence, and finally the attribution of the sentiment based on the method followed, the visualization and the interpretation of the results. Regarding the sentiment analysis technique, there are two axes: the process based on machine learning and the one established in a lexicon. The hybrid method combines the primary two techniques (43).

The core of sentiment determination is distinguished in a binomial characterization, positive or negative. There can also be a third category, i.e., positive, negative or neutral emotional content. Essentially, with the machine learning methods, the problem of the sentiment attribution of comments has resulted in a data categorization problem. By extension, classification algorithms are usually applied, such as, among others, Logistic Regression (LR), Support Vector Machine (SVM), Random Forest (RF), Stochastic Gradient Descent (SGD), and ensemble classifiers.

Logistic regression can be applied to classification tests by predicting the binary dependent variable from a set of independent variables. Another binary, non-probabilistic classifier is the support vector machine which relies on kernel mapping. Moreover, the random forest algorithm produces multiple trees where each one of them is constructed using a random subset of the vector features. The decisions of each tree are synthesized utilizing an algorithm that gives the outcome. Additionally, two class machine learning classifiers implemented in this study which are: the two class neural network, the two class Bayes point machine, the two class boosted decision tree, the two class averaged perceptron, the two class decision jungle, the two class local deep SVM, and all these algorithms develop a binary classification model (44, 45).

The overall statistical conduct of machine learning classifiers is appraised with the aid of respective parameters, the most popular being the accuracy (1), precision (2), recall (3), and F1 score (4). These metrics are composed of TP, TN, FP, and FN values representing true positive, true negative, false positive, and false negative values in a produced confusion matrix (42, 44). The equations of the evaluation parameters are:


(1) $$\begin{array}{l} Accuracy=\frac{TP+TN}{TP+TN+FP+FN} \end{array}$$

(2) $$\begin{array}{l} Precision=\frac{TP}{TP+FP} \end{array}$$

(3) $$\begin{array}{l} Recall=\frac{TP}{TP+FN} \end{array}$$

(4) $$\begin{array}{l} F1\;score=2^{*}\frac{Precision^{*}Recall\;}{Precision+Recall} \end{array}$$

Natural language processing (NLP) is a subfield of computational science and refers to a software's capability to automatically manipulate and classify information using natural language, like text and speech. Sentiment analysis was based on a Natural Language Processing algorithm, a subset of Artificial Intelligence (AI). Contemporary Natural Language Processing methods, like sentiment analysis classifiers and models, have been successfully utilized to fight the COVID pandemic (45).

### 2.2. Methodology applied in the case study

A machine learning technique was performed with the Azure Excel Add-in to classify Facebook data (comments) from the official page of EODY. In particular, daily reports publicized from November 2021 to January 2022 were collected. Moreover, after processing 300 comments, positive, negative and neutral sentiments were included. This approach involved analyzing the comments of the people discussing the daily EODY reports on Facebook, which included, among others, the number of deaths, number of patients in the critical condition/intensive care, patient gender, and number of COVID-19 confirmed cases based on rapid and molecular tests (46, 47).

The Chi square independence test was used to evaluate sentiment orientation across gender and month. That is, to examine whether Facebook comment frequencies significantly differed across men and women and across the three months of the last winter of the pandemic in Greece, namely, November 2021-January 2022. The null hypothesis in each case is that the two genders do not comment independently of one another. Furthermore, the sentiment orientation of the comments varies somewhat regarding the month they appeared. Results with a *p*-value <0.05 were considered statistically significant.

**Figure 2:**
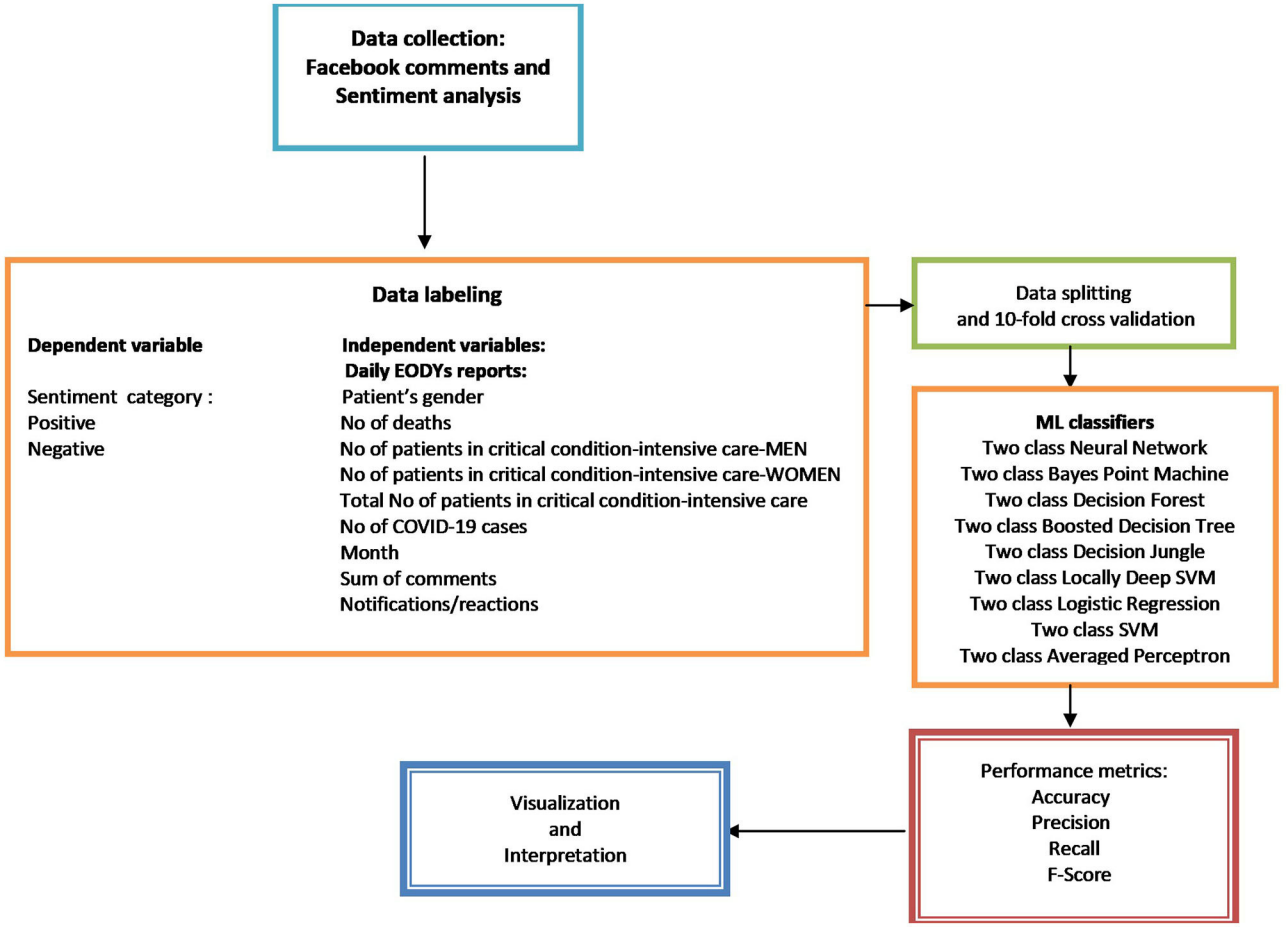
Data collection and research workflow.

In the next phase, all neutral comments were omitted, and the remained comments were corrected manually to leverage the machine learning performance, especially in the case of sarcasm and irony in Greek phrases. Finally, only the positive and negative comments were selected to create and compare machine learning classifiers, namely 199 comments. Subsequently, models were developed in Microsoft Machine Learning Studio (Classic) to classify public sentiments, positive or negative, based on each surveillance report on EODY's official Facebook page (Figure 2). Statistical analysis was performed by SPSS v.27 statistical software (SPSS Inc., USA) and Microsoft Excel (48).

This research tests nine classification techniques and the respective evaluation metrics, namely two-class classifiers: Neural Network, Bayes Point Machine, Decision Forest, Boosted Decision Tree, Decision Jungle, Locally Deep Support Vector Machine, Logistic Regression, Support Vector Machine and Averaged Perceptron (Figure 2). Moreover, cross validation techniques strengthen the part of the development of mathematical models and avoid overfitting or underfitting problems. This technique is followed in machine learning to evaluate the reliability of a model trained from a dataset and control data variability (49).

Specifically, the K-fold cross validation technique was adopted because it is one of the most common approaches (50–54). The model is trained using an exclusive combination of K-1 subsets of data and tested on the remaining subset. Briefly, the training dataset is divided into K subsets of equal size, which in this study equals ten. Subsequently, ten models will be generated for each subset of training data and evaluated by averaging the performance metric values of the models, i.e., accuracy, precision, recall and F1 score^3^ (Figure 2).

## 3. Results and discussion

**Figure 3:**
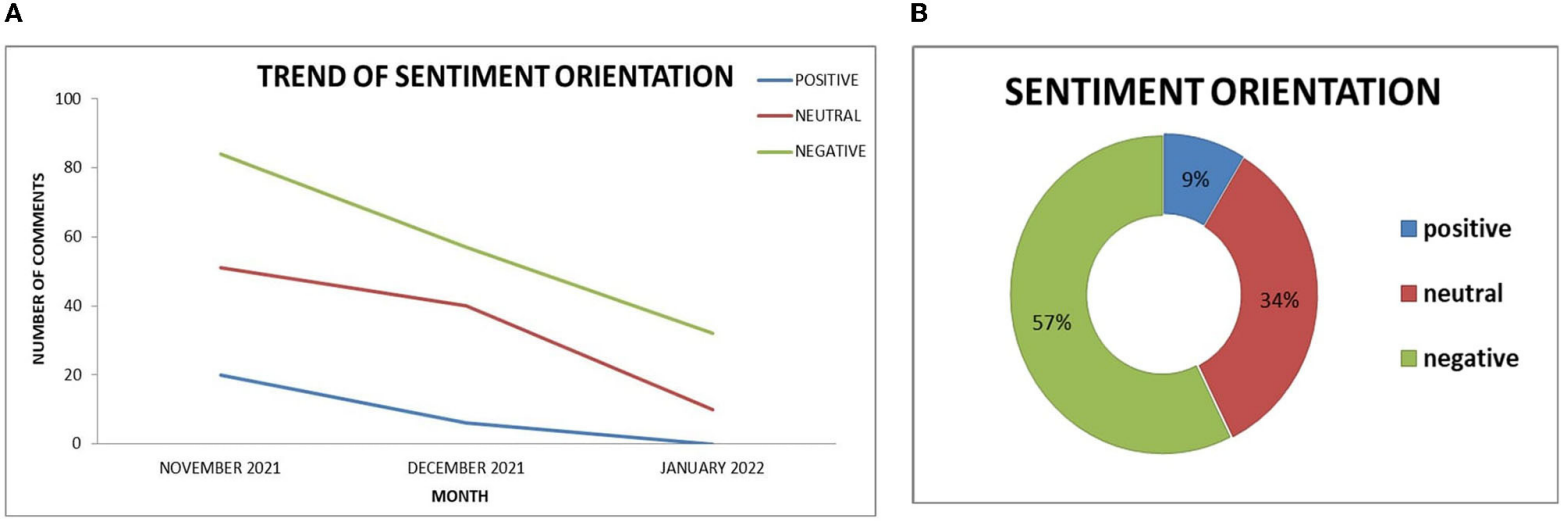
**(A)** Trend of comment's sentiment orientation. **(B)** Percentage of sentiments classes.

The text analysis revealed positive, negative and neutral sentiments expressed on EODY's Facebook page in 3 months, from November 2021 to January 2022 (Figures 3A, B). The vast majority of the sentiments have a negative orientation, with the respective percentage rising to 57%. On the contrary, 34% neutral and 9% positive comments were also documented (Figure 3B).

A declined trend of negative, neutral and positive sentiments at the end of the winter, in January 2022, is also highlighted in Figure 3A. The interpretation of this downward trend of all types of comments stems from the revision of EODY's dissemination policy. More concretely, the organization decided to publish weekly epidemiological reports of COVID-19 during the first ten days of January instead of the daily publication of the records. Consequently, the public lost interest, and citizens' comments on the EODY Facebook page decreased.

The variation in sentiment orientation depends on the course of the pandemic and the corresponding pandemic waves that contribute to the public's positive or negative comments (25). A related study reported sentiment analyses of COVID-19 Facebook posts from January 1, 2019, to March 18, 2020. More precisely, comments were collected from three Facebook public health agencies' pages, the Ministry of Health in Singapore, the Centers for Disease Control and Prevention in the United States, and Public Health England in England. A negative polarity was indicated for most of the comments in all three public agencies. Moreover, the temporal analysis showed variations between the number of posts in three countries, partly explained by the use of Facebook by the health services to publicize information and topics on COVID-19 and other health issues (55).

Similarly, the high negative percentage of public opinion during the pandemic was in line with other research outcomes; namely: averaged positive, negative, and neutral sentiments were at 58%, 22%, and 17% in the United Kingdom, 56%, 24%, and 18% in the United States, respectively (56).

Table 1 highlights the sentiment proportion of each comment for every month of the examination period, namely November 2021, December 2021 and January 2022. Approximately half of the Facebook posts (51.7%) were formulated in November, while the lowest percentage of comments was documented in January. Overall, January noted the lowest percentage of Facebook posts in all three categories, mainly positive (0%), negative comments (10.7%), and neutral (3.3%).

**Table 1:** Percentage of sentiments per month.

Sentiment^*^Month^1^	**November**	**December**	**January**
Negative	28.0%	19%	10.7%
Neutral	17%	13,3%	3.3%
Positive	6.7%	2%	0.0%
Total	51.7%	34.3%	14%

1 X^2^ = 12,786, df = 4, *p* < 0.05.

* Correlation.

Furthermore, it is worth noting that the continuous decrease in the number of posts throughout the 3 months was statistically significant (*p* < 0.05). As mentioned previously, this decline can be attributed to the decision made by EODY (National Public Health Organization in Greece) to change the frequency of publishing COVID-19 surveillance reports from daily to weekly. This alteration in the reporting schedule directly impacted the quantitative production of Facebook posts among the public, reducing overall activity on the platform over time.

During the initial phase of the pandemic crisis in China, specifically between mid-January and mid-February, there was a noticeable rise in negative comments on a social networking platform. This increase could be attributed to the official confirmation and documentation of human-to-human virus transmission during that period. The revelation of this critical information likely impacted public sentiment, leading to a surge in adverse reactions and discussions on the platform. A survey conducted on the time trend of 500 tweets based on Vader Lexiconin in August 2021 found that neutral comments accounted for 20% of the total, followed by negative comments at 17% and positive comments at 15%. However, in September of the same year, there was a slight increase in positive comments by 2%. On the other hand, the percentages of neutral and negative comments remained relatively stable, with no significant changes compared to the previous month (27, 57).

The sentiment patterns varied between genders. Specifically, men (31.3%) tended to make negative comments more than women (26.3%). Moreover, men expressed positive comments nearly three times more frequently than women. In summary, the data indicates that men, in general, contribute a higher number of comments compared to women. Moreover, men tend to produce a greater proportion of negative comments compared to women. However, when it comes to neutral comments, the percentages are relatively similar, with men having a slight predominance.

Women demonstrated a higher frequency of engaging with the healthcare system and had different evaluations of the health system regarding the COVID-19 crisis during the examination period in Greece (as shown in Table 2). Interestingly, the sentiment orientation of comments on EODY's Facebook page was not significantly influenced by gender (*p* > 0.05).

**Table 2:** Percentage of sentiments per gender.

Sentiment^*^Gender^1^	**Men**	**Women**
Negative	31.3%	26.3%
Neutral	17.0%	16.7%
Positive	6.3%	2.3%
Total	54.7%	45.3%

1 X^2^ = 4,273, df = 2, *p* > 0.05.

* Correlation.

Similar findings were observed when examining gender differences in expressing positive and negative sentiments on a health web forum. For instance, females used positive words such as “thank” and “glad” twice as often as men, while negative words like “problem,” “scary,” and “illness” were also used twice as frequently by females. On the other hand, males used positive words such as “important” and “receptive” more regularly, and negative words like “issue,” “fever,” and “aches” were used twice as often by males (42, 47). According to the survey results, it was found that men sent a higher number of messages compared to women. Specifically, men accounted for ~40% of the total messages analyzed, whereas women constituted around 27% of the messages. This discrepancy in message participation between genders was observed in a dataset of nearly 23,000 messages (42).

**Figure 4:**
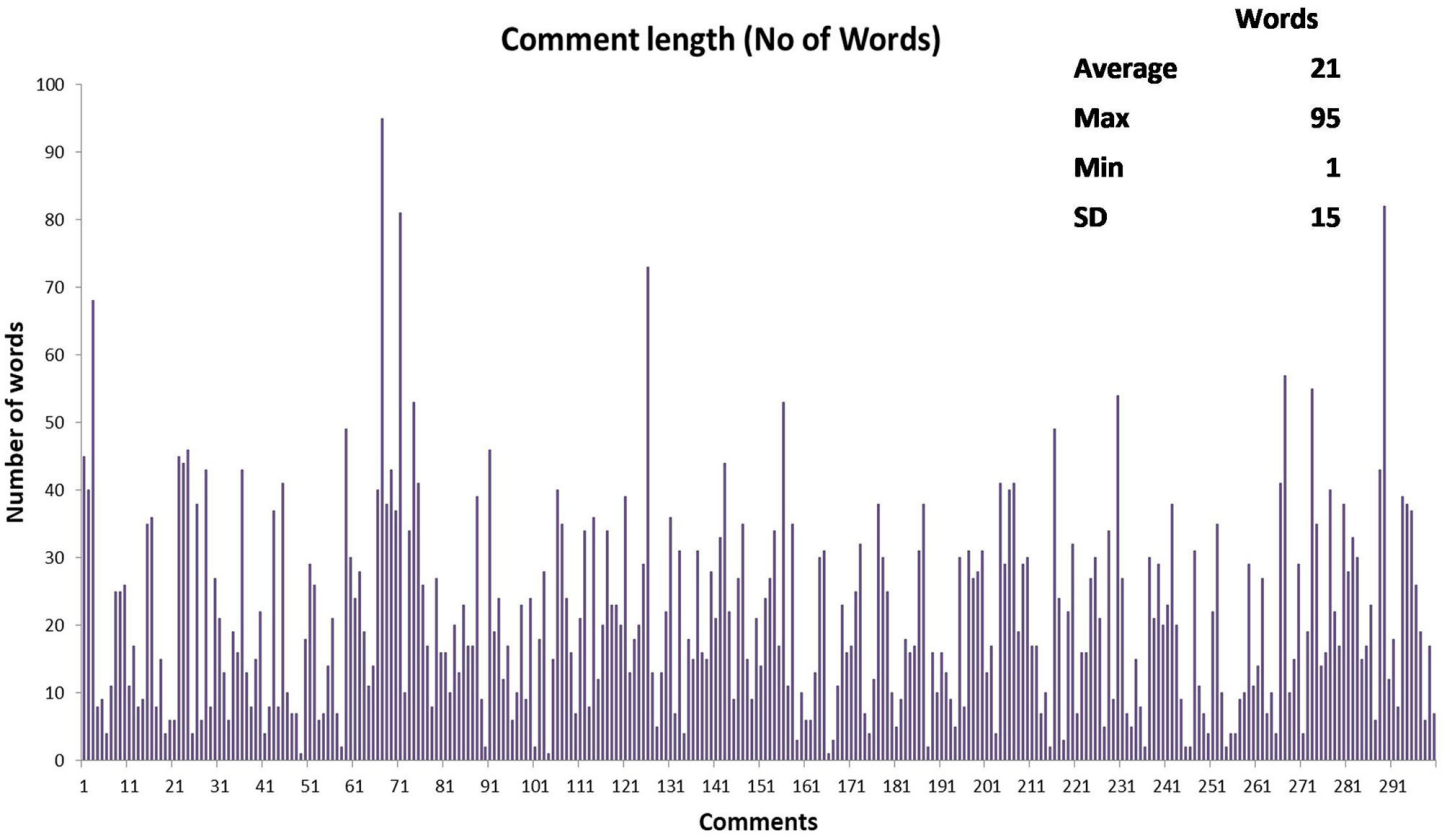
Number of words per comment.

Figure 4 outlines the number of words composed for each comment. In total, 300 Facebook posts were collected from the EODY page. One comments consisted of one word, while one comment comprised of 95 words. The average post length was 21 words.

**Figure 5:**
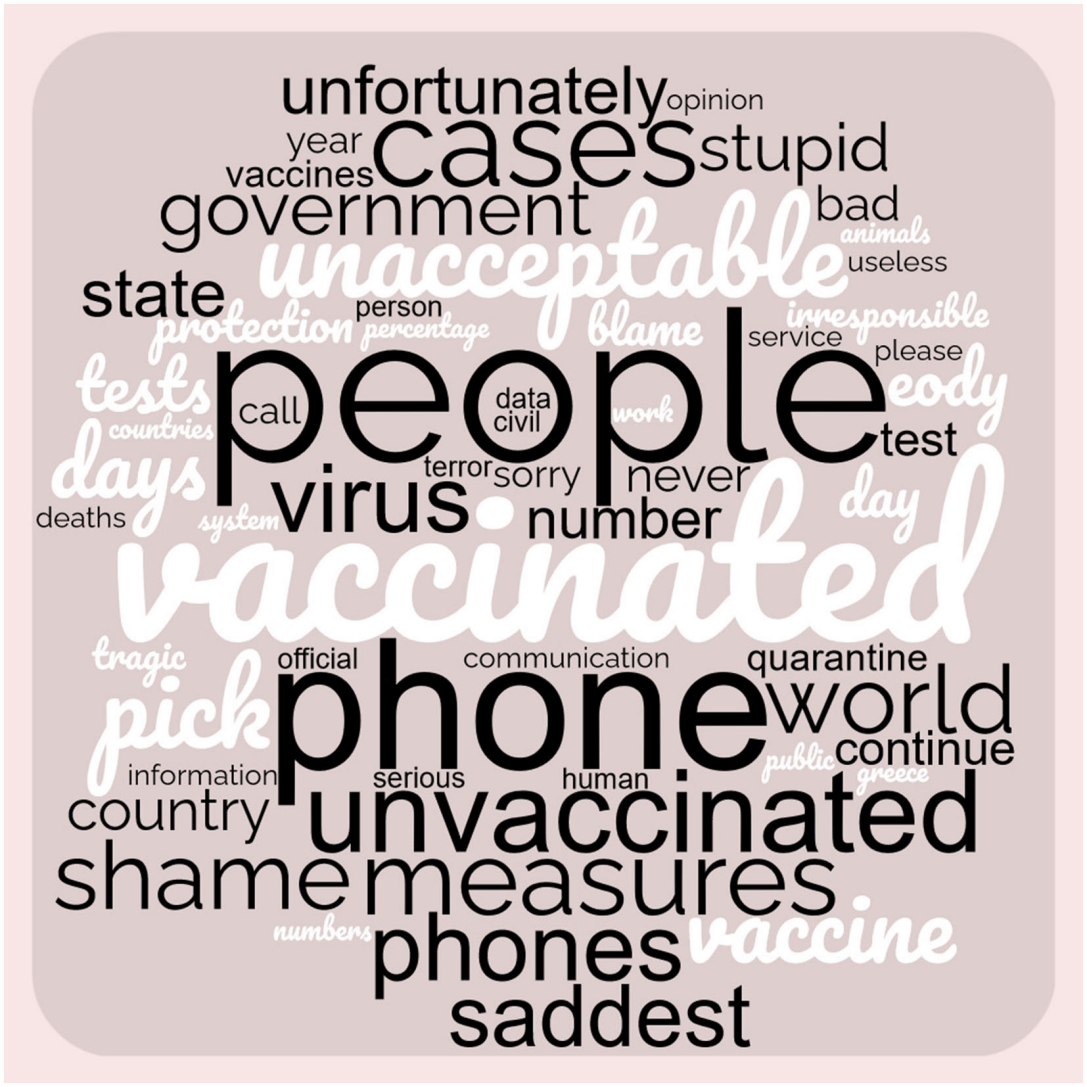
The word cloud represents the frequency of word occurrence and serves as a depiction and evaluation of the audience's perspectives, public perceptions, and documentation thereof (59 words).

Figure 5 illustrates the words with a higher frequency rate in the public comments, reflecting opinions and debating issues, the greater the word the higher the frequency. Overall, 59 words are depicted. Words with the highest frequency of occurrence include “government,” “vaccinated people,” “unvaccinated,” “telephone communication,” “health measures,” “virus,” “COVID-19 rapid/molecular tests,” “sad” and as expected, “COVID-19.” Additionally, the quality of public data contained in the epidemiological reports published by EODY, as well as the responsibility toward the direction of reduction of coronavirus cases, are also the focus on citizens' debate.

The public discussed topics related to the lack of communication and guidance to people infected with COVID-19, especially on the first day after the positive result of a rapid or molecular test. During the pandemic, words like COVID, vaccines and all the related derivates like corona, pandemic, infection, test, and measures are some of the most widely discussed. In the same context, the pandemic waves modified the terms that were commented among the public. People were interested in the virus early in the COVID-19 pandemic. Afterwards, public opinion focused on government measures, hygiene, and social and financial terms (27). The communication strategy of EODY and social media activity on Facebook are also criticized, while transparency issues emerged (58, 59).

Along the same line, public health and physical distancing are among the ten most discussed topics during WHO's press conferences during the pandemic. In addition, other hot topics like vaccine manufacture, contact tracing, report case, mild case, severe diseases, vaccination coverage, social measure, global solidarity, health emergency programme, disease control, and lock-down were underlined. Transparency issues also emerged during a survey regarding the UK government's COVID-19 control strategy in the first wave (April 2020). Citizens with various demographic and socioeconomic backgrounds were skeptical about the justification of opacity due to the pandemic, mistrust of politics, scientific evidence about the pandemic, the communication strategy and the decision-making processes implemented by the officials (60).

The word cloud analysis revealed that specific terms unrelated to sentiments appeared prominently, indicating the topics widely discussed among the public. One notable topic was vaccines and vaccinations, including discussions on public hesitancy, knowledge gaps, and misinformation. Another unique pattern was that younger individuals prioritized vaccination as a top concern compared to the older population, who relied on their previous vaccination experiences for other diseases. Transparency in government actions, vaccine manufacturing, and the lack of public trust, particularly among those associated with the anti-vaccination movement, were also significant issues. These findings emphasize the importance of the government's effective communication management and strategic planning to address these concerns and build public trust (61–65).

In a separate survey conducted on the impact of COVID-19 in India, a country heavily affected by the pandemic, similar words such as “COVID” and “stay home” were observed. Additionally, topics related to hashtags such as #Lockdown, #COVID, and #corona were frequently discussed. The survey also revealed discussions on issues about working from home. These findings indicate that specific themes and concerns were shared across different studies conducted in different regions, highlighting the global impact and shared experiences related to the COVID-19 pandemic (66).

An analogous study aimed to analyze discussion topics from March 7 to April 21, 2020, and to perform sentiment analysis on 4 million Tweets. This probe revealed that people were interested in issues related to the confirmed cases and death rates, health authorities and government policies and adverse psychological reactions or psychological consequences (62). Negative emotions during the pandemic, such as anger, fear or sadness, were also justified in a study that determined the sentiment orientation at the global level from tweet posts from January 28 to April 9, 2020. The findings indicated that public health agencies should include measures toward leveraging citizens' negative emotions and implement new actions in general hygiene communication management (67).

One aspect recognized during the COVID pandemic is mental health because religion and the spiritual factor are resilient factors. A research work underlined that factor with the aid of interviews and natural language algorithms. The outcome spotlighted the positive effect of religion in human resilience during the symptoms of the disease. Moreover, words like security, confidence, tranquility, and peace were among the most stated between groups (68, 69).

**Figure 6:**
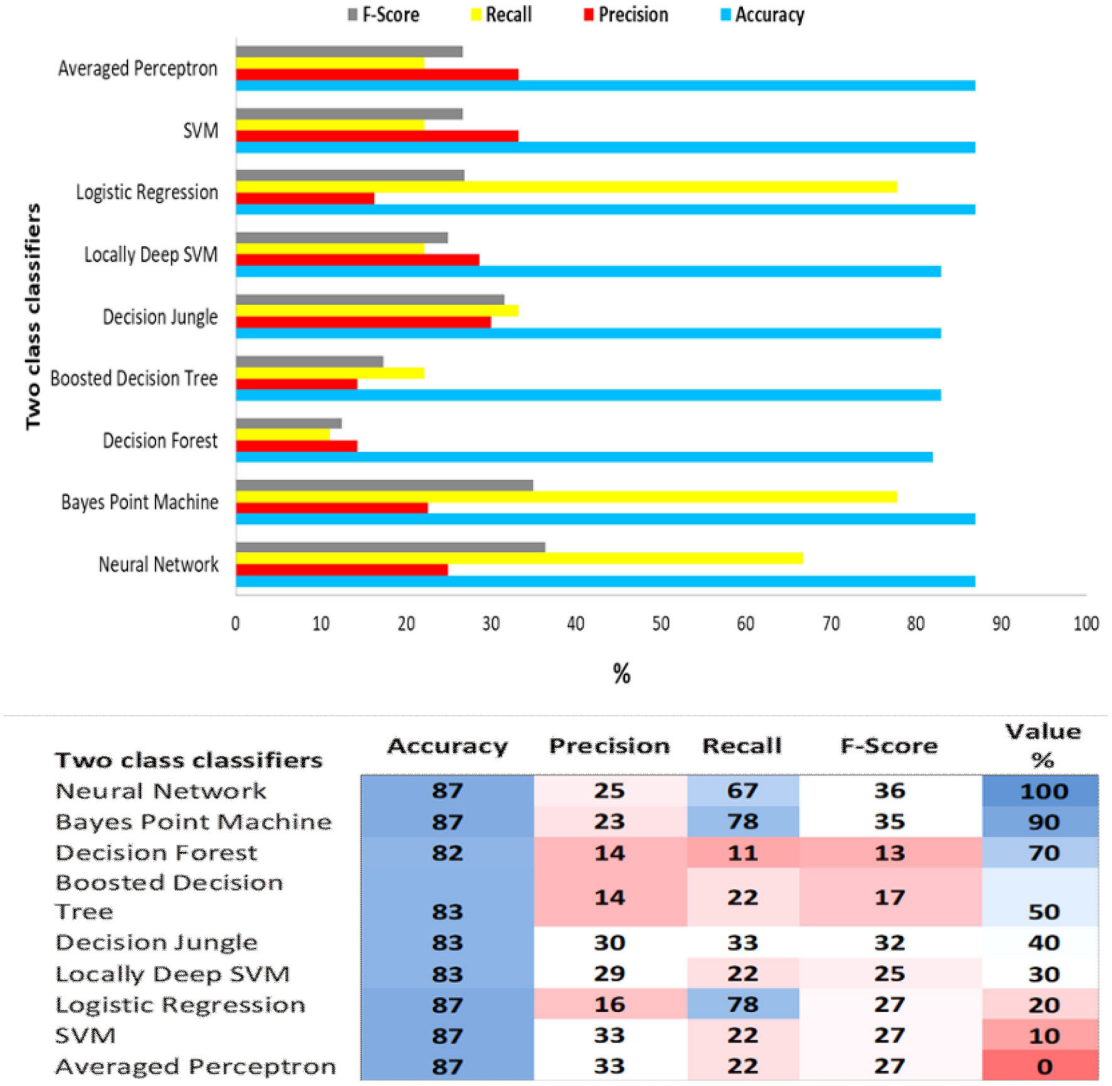
Evaluation of two class classification machine learning algorithms and the performance heat map.

In Figure 6, the comparison of the proposed classifiers is outlined. Two class Neural Network and Bayes Pointt Machine, were found to perform better than the other classifiers. Particularly, these two had the same score in Accuracy (87%), F1 score (36 and 35%), Precision (25 and 23%) and Recall (67 and 78%). The next classifier with the best score was the Logistic Regression classifier with 87%, 27%, 16%, and 78% metric evaluation values, respectively.

Other metrics should also be considered to estimate the predictive models' performance. The precision value for a class is the number of true positives divided by the total number of items marked as belonging to the positive class. Recall in this context is defined as the number of true positives divided by the total number of items that belong to the positive category. In our analysis, where positive sentiment has been labeled as a “positive” and negative sentiment as “negative”, it is illustrated that the lowest Precision value is achieved by the Decision Forest and Boosted Decision tree, while the highest, 33%, by the Averaged Perceptor and the SVM. In other words, these last two algorithms have the best predictive value of positive emotions.

The highest Recall score was performed from the Logistic Regression and Bayes Point Machine classifiers, meaning that these two algorithms are more sensitive than the other classifiers. Finally, the value of F1 shows that the Neural Network and Bayes Point machine have the best performance, 36% and 35%, respectively, because the F1 metric value score combines the sensitivity and specificity of the considered algorithms.

An additional research study also justified the superiority of the Support Vector Machine classifier in sentiment analysis regarding public health issues. This specific research validated the accuracy of machine learning algorithms during a collection of English posts from Instagram during the pandemic 70.

A similar study applied machine learning models for sentiment analysis. It proved that the multilayer perceptron and the support vector machine algorithm had the best evaluation score, with 76% and 74% accuracy, respectively (25).

A further study confirmed that Linear Regression, Random Forest, and Decision Tree classifiers achieved an excellent accuracy score, while the accuracy of the Support Vector Machine was 95%. After cross validation of the models used in this research, the Support Vector Machine and Random Forest model proved sufficient (70). One more research conducted in Jordan considered Facebook posts highlighted the advanced performance of Support Vector Machine in alignment with a second algorithm, the Whale Optimization. The accuracy score ranged from 69,05% to 84,64% in various datasets (28).

In the same context, an extra research paper proposed a deep learning model for sentiment analysis and classification regarding two datasets of tweets from January 2019 to March 2020 and December 2019 until May 2020. The best accuracy results were succeeded with the Logistic Regression, which is in line with the results of our research, and the Random Forest classifiers reaching up to 75% and 81%, respectively 72.

Users' satisfaction levels toward governmental mobile applications in Saudi Arabia and respective sentiment analyses were obtained from another inquiry (71). Five machine learning classifiers were implemented: random forest (RF), bagging, support vector machine (SVM), logistic regression (LR), and naïve Bayes (NB). They revealed that the Support Vector Machine achieved the best accuracy score (94.38%) implementing the SMOTE technique. In line with this study, the researchers conducted a sentiment analysis of users' opinions of mobile Apps that the Saudi Arab government introduced to combat the pandemic. They reported that the K-Nearest Neighbor and Decision Tree classifiers outperformed in terms of accuracy by 78% and 60%. Support Vector Machine and Naïve Bayes classifiers accomplished 55% and 51% accuracy scores (72, 73).

The exploitation of social network data to develop reliable early information surveillance and warning system for pandemic outbreaks resulted in a three-part integrated system. Twitter extracted data were tested for sentiment classification using various classifiers that outlined the spatiotemporal dimension in the early period of the COVID-19 outbreak. A version of the Decision Tree Classifier outperformed conventional sentiment and geolocation classification models, achieving 94.3% and 80.8% Accuracy values, respectively (74). In our sentiment analysis, Decision Forest and Decision Jungle classifiers scored Accuracy values over 80%.

Precise sentiment classification is vital to accurate predictions on infection disease evolution. In this line, sentiment analysis at the word and document level was performed using two machine learning algorithms. Accuracy values were equivalent to ~87% and 92%, aligning with our research results since all algorithms' corresponding accuracy metrics ranged between 83% and 87% (75).

Our literature search was limited to peer-reviewed publications in English, indexed in the Scopus database in the Related Work section. The choice of databases and keywords for the literature search impacted the number of studies selected for this study. Some data source biases are inherent to social media, such as authentication issues of the user profile. Other biases that researchers could have accounted for were posts generated from bots and non-individual accounts. Also, the data set could be more extended, but extracting comments from Facebook is relatively more complex than other platforms, although Facebook is the most popular platform in Greece. Topics of sarcasm, irony, informal expressions, humor, and slang are challenging to detect by computer programs (49, 76, 77). In addition, a large data set could improve the accuracy of our results. Another limitation of our study is the lack of an occurrence map that considers the words appearing in the word cloud (Figure 5). Such a map could provide insights into the public's perception and topics of discussion, allowing for the conceptualization and determination of the connections between sentiment, topics, and opinions on public health issues.

Despite the limitations mentioned above, this study has presented a case study conducted in Greece, which identifies key areas that should be taken into account by researchers, health professionals, health organizations, and crisis communications managers. Future initiatives will examine the performance of additional machine learning techniques. Also, a more considerable scale evaluation will be performed to provide a more complete insight into the evaluation metrics of the algorithms.

## 4. Conclusion

Considering that the COVID-19 pandemic crisis is still a major concern to governmental and public agencies, sentiment analysis can provide a better insight into the ongoing side effects in social terms, as it can predict the positive, negative, and neutral orientation of citizens. The global increase and broad use of Social Networking Sites have initiated new forms of expressing opinions and sentiments on SNS like Twitter, Facebook, etc.

This research analyzed the public perception and sentiment orientation regarding the daily surveillance reports of EODY in Greece. The respective sentiments were classified into three main comment categories: positive, neutral, and negative. Moreover, this study implemented machine learning algorithms to predict public positive or negative sentiment orientation concerning Facebook and COVID-19 data from daily surveillance reports. Furthermore, this research study covered a wide range of sentiment analysis approaches from the state of the art classifiers and compared each performance in terms of accuracy, F1 score, recall and precision. Overall, two classifiers, namely two class Neural Network and two class Bayes Point Machine, achieved high sentiment analysis accuracy and F1 score, particularly 87% and over 35%.

Based on the results, sentiment analysis and prediction models can provide critical supplementary information about the expression of sentiments during the COVID-19 pandemic. Gender and time are two factors that may determine public opinion on medical topics and especially those regarding the pandemic crisis. Overall, further research is required to advance algorithms and predicting models for sentiment and opinions to help monitor public health services and decision making processes during the pandemic.

In future work, new extended sentiment analysis could be created by implementing new classifications, considering more generated data from Greek Social Networking Sites, and keeping the current study up to date. Thus, it could extend prospective sentiment analysis using multiple models and provide additional studies to interpret and manage the outcomes.

## Author contributions

Conceptualization: CS and EG. Methodology: CS. Data curation: AT. Writing—original draft preparation: KK, CS, and CK. Writing—review and editing: EN, CT, and YK. Supervision: EC and AC. Project administration: EB and ID. All authors have read and agreed to the published version of the manuscript.

## Footnotes

^1^ https://search.cdc.gov/search/?query=syndromic%20surveillance&dpage=1

^2^ https://www.vosviewer.com/

^3^ https://learn.microsoft.com/en-us/azure/machine-learning/component-reference/cross-validate-mode
