# Supplementary material for: Sentiment analysis of epidemiological surveillance reports on COVID-19 in Greece using machine learning models
Source: Front Public Health. 2023 Jul 18;11:1191730. doi: 10.3389/fpubh.2023.1191730 (PMC10392838; doi:10.3389/fpubh.2023.1191730)
Supplement: Supplementary file 1 [file Data_Sheet_1.pdf]

Supplementary Material

Sentiment Analysis of Epidemiological Surveillance Reports On COVID-19 In Greece Using Machine Learning Models

Christos Stefanis, Elpida Giorgi, Konstantinos Kalentzis, Athanasios Tselemponis, Evangelia Nena, Christina Tsigalou, Christos Kontogiorgis, Yiannis Kourkoutas, Aikaterini Chatzaki, Ioannis Dokas, Theodoros Constantinidis and Eugenia Bezirtzoglou*

*** Correspondence:** Eugenia Bezirtzoglou:empezirt@yahoo.gr

Supplement 1. Sentiment analysis and public health

a) The concept of sentiments analysis in public health

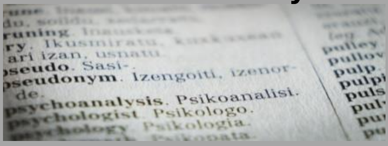

computational linguistics

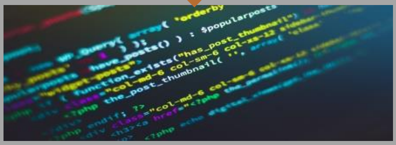

natural language processing

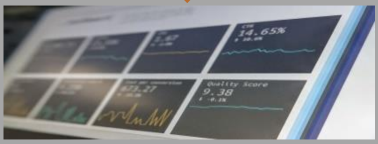

sentiment analysis

b) conceptual analysis and science mapping (co-occurrence analysis)

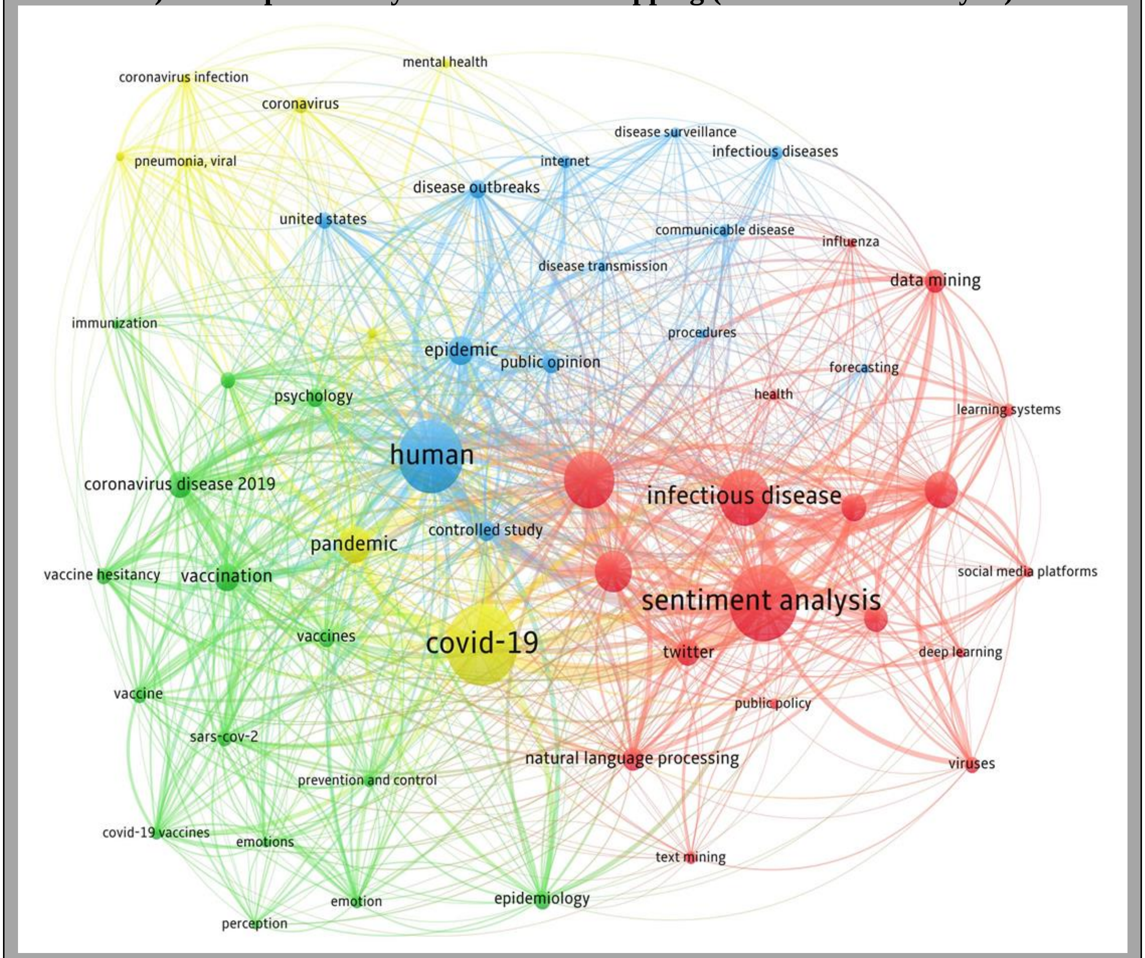

c) sentiment analysis, work applied and applications

Sentiment analysis applications	Work applied	Research paper
• monitoring mass gatherings during a pandemic	Mobile-Health applications	A Proposed Framework for DevelopingUser-Centred Mobile Healthcare Applications for the Biggest Annual Mass Gathering (Hajj) Post COVID-19
• estimate disease incidences for the current week (nowcasting) onsidering the social media data and the disease case counts reported by the Government agencies	Digital epidemiology/disease epidemics	A Social Media Time-Series Data Analytics Approach for Digital Epidemiology
• sentiment changes in COVID-19-related tweets and public health policies and events	Build a surveillance system for monitoring people’s attitudes towards public health policies	Analyzing Twitter Data to Evaluate People’s Attitudes towards Public Health Policies and Events in the Era of COVID-19
• measure the spatio-temporal sentiment towards a new vaccine	Measuring population health behaviors over time and space	Assessing Vaccination Sentiments with Online Social Media: Implications for Infectious Disease Dynamics and Control
• extract knowledge regarding infectious diseases, their symptoms, or poor environment conditions what promote the propagation of these diseases	CollaborativeHealth, an infodemiology platform	CollaborativeHealth: Smart echnologies to Surveil Outbreaks of Infectious Diseases Through Direct and Indirect Citizen Participation
• identify and extract disease symptoms and their associations	Biomedical text analytics system, DiseaSE (Disease Symptom Extraction)	DiseaSE: A biomedical text analytics system for disease symptom extraction and characterization
• user behavioral patterns on the web	A social media based mosquito-borne disease surveillance and outbreak management	Effective surveillance and predictive mapping of mosquito-borne diseases using social media
• predict infectious disease such as influenza-like illness (ILI) outbreaks using Twitter data	Infectious Disease Prediction	Evaluation of IBM Watson Natural Language Processing Service to predict influenza-like illness outbreaks from Twitter data
• public sentiment related to COVID-19 vaccines	Real-time analysis of large bodies of text related to disease outbreaks and vaccination	Fine-tuned Sentiment Analysis of COVID-19 Vaccine–Related Social Media Data: Comparative Study
• identifies and characterizes user-generated messages related to opioid abuse, heroin injection drug use, and HIV status	Infoveillance	Identification and characterization of tweets related to the 2015 Indiana HIV outbreak: A retrospective infoveillance study
• identifying the infectious or recovered period of flu cases through social media	Epidemic control and prevention in real time	Infectious or Recovered? Optimizing the Infectious Disease Detection Process for Epidemic Control and Prevention Based on Social Media
• detect the emergence of diseases, particularly influenza-like illnesses, and foster disease surveillance	Disease classification	Influenza-like Illness Detection from Arabic Facebook Posts Based on

systems		sentiment Analysis and 1D convolutional Neural Network
• information-seeking patterns during pandemics such as COVID-19 using Google Trends	Infodemiology	Information-Seeking Patterns During the COVID-19 Pandemic Across the United States: Longitudinal Analysis of Google Trends Data
• disease outbreaks that occur overseas avian influenza A(H7N9)	Disease surveillance and public sensing	Leveraging social networking sites fordisease surveillance and public sensing: the case of the 2013 avian influenza A(H7N9) outbreak in China
• content analysis and information surveillance	Infodemiology	Main uses of Instagram in oral health research–A scoping review
• patient online review	Fading topics and sentiment trends in physician rating websites	Mining topic and sentiment dynamics in physician rating websites during the early wave of the COVID-19 pandemic: Machine learning approach
• the spread of influenza can be predicted with high accuracy<br>• monitor the spread of influenza in selected cities in real-time.	Detecting and monitoring diseases in real time	Mining Twitter Data For Influenza Detection and Surveillance
• social, medical, public health and technology sciences.	Vaccine hesitancy	Multi-perspectives systematic review on the applications of sentiment analysis for vaccine hesitancy
• predict the possible number of cases with H1N1 disease	Integrated Disease Survellience Program	Prediction of Influenza-like Illness from Twitter Data and Its Comparison with Integrated Disease Surveillance Program Data
• drug abuse epidemiology	Development of a novel semantic web platform	PREDOSE: A semantic web platform for drug abuse epidemiology using social media
• weekly flu rate predictions	Track disease outbreaks and provide early warnings, even for newest outbreaks	Preliminary Flu Outbreak Prediction Using Twitter Posts Classification and Linear Regression With Historical Centers for Disease Control and Prevention Reports: Prediction framework Study
• emotional response of Moroccan citizens to COVID-19 pandemic and its effects	Decision-making assistance tool for COVID-19 mitigation and management	Real-Time Infoveillance of Moroccan Social Media Users' Sentiments towards the COVID-19 Pandemic and Its Management
• identify emotions in social media conversations about COVID-19	Public health surveillance	Rise and fall of the global conversation and shifting sentiments during the COVID-19 pandemic
• identify the locations of disease outbreaks	Public health surveillance	Sentiment Analysis as a Service: A social media based sentiment analysis framework
• sentiment analysis to document patients' experience and emotional distress of dermatological diseases (alopecia areata (AA), idradenitis suppurativa HS), and psoriasis (PsO) in comparison to fibromyalgia	Identify patients' experiences of skin disease	Sentiment analysis of tweets on alopecia areata, hidradenitis suppurativa, and psoriasis: Revealing the patient experience

(FM)		
• reporting of SARS-CoV-2 outbreak status	Develop reliable early information surveillance and warning system for pandemic outbreaks	Sentiment-Based Spatiotemporal Prediction Framework for Pandemic Outbreaks Awareness Using Social Networks Data Classification
• management of the pandemic and its waves might actually represent a novel preventive approach to hinder emotional contagion, disseminating reliable information and nurturing trust	COVID-19 emotional contagion surveillance	Surveilling COVID-19 Emotional Contagion on Twitter by Sentiment Analysis
• flu prediction/detection	Flu disease surveillance systems	Text Classification of Flu-related Tweets Using FastText with Sentiment and Keyword Features
• surveillance of mosquito-borne diseases<br>• disease classification with demographic variables<br>• detection prediction<br>• public awareness	Decision making-mosquito surveillance programs	Text mining in mosquito-borne disease: A systematic review
• identification of the main topics posted by Twitter users related to the COVID-19 pandemic.	Infoveillance	Top Concerns of Tweeters During the COVID-19 Pandemic: Infoveillance Study
• Identification of dominant themes, topics, sentiments, and changing rends in tweets about the COVID-19 pandemic	Infoveillance	Topics, Trends, and Sentiments of Tweets About the COVID-19 Pandemic: Temporal Infoveillance Study
• detecting influenza epidemics using Twitter	Infectious Disease Prediction and early warning	Twitter Catches The Flu: Detecting Influenza Epidemics using Twitter
• public concern about epidemics	Monitoring public health concerns	Twitter sentiment classification for measuring public health concerns
• surveillance	Using Twitter as a means of surveillance for public health crises and specifying groups or populations at risk	Using a mixed methods approach to identify public perception of vaping risks and overall health outcomes on Twitter during the 2019 EVALI outbreak
• produce national flu forecasts for the United States	Influenza forecasting using electronic health records (EHR) and in Internet users' search activity	Using electronic health records and Internet search information for accurate influenza forecasting
