# Supplementary material for: Sentiment analysis of epidemiological surveillance reports on COVID-19 in Greece using machine learning models
Source: Front Public Health. 2023 Jul 18;11:1191730. doi: 10.3389/fpubh.2023.1191730 (PMC10392838; doi:10.3389/fpubh.2023.1191730)
Supplement: Supplementary file 2 [file Data_Sheet_2.pdf]

Supplementary Material

Sentiment Analysis of Epidemiological Surveillance Reports On COVID-19 In Greece Using Machine Learning Models

Christos Stefanis, Elpida Giorgi, Konstantinos Kalentzis, Athanasios Tselemponis, Evangelia Nena, Christina Tsigalou, Christos Kontogiorgis, Yiannis Kourkoutas, Aikaterini Chatzaki, Ioannis Dokas, Theodoros Constantinidis and Eugenia Bezirtzoglou*

*** Correspondence:** Eugenia Bezirtzoglou:empezirt@yahoo.gr

Supplement 2. Co-occurrence analysis

id	label (item)	cluster	weight <Links>	weight<Total link strength>	weight <Occurrences>	score <Avg. pub. year>
82	attitude to health	2	37	85	9	20.175.556
178	communicable disease	3	32	64	7	20.192.857
213	controlled study	3	41	110	12	20.194.167
219	coronavirus	4	27	47	9	2021
221	coronavirus disease 2019	2	40	179	16	2.021.125
222	coronavirus infection	4	25	58	5	2020
233	covid-19	4	50	306	58	20.213.103
239	covid-19 vaccines	2	27	82	7	20.217.143
268	data mining	1	28	89	14	20.196.429
280	deep learning	1	23	39	6	20.208.333
319	disease outbreaks	3	40	106	11	20.178.182
321	disease surveillance	3	26	47	6	20.173.333
322	disease transmission	3	25	42	6	20.133.333
325	diseases	1	31	91	16	2.018.875
365	emotion	2	28	76	7	20.205.714
374	emotions	2	26	51	5	2020
390	epidemic	3	47	160	18	2019
396	epidemiology	2	33	84	11	20.214.545
437	forecasting	3	22	31	5	2020
471	health	1	23	34	6	20.161.667
532	human	3	52	387	51	20.177.647
548	immunization	2	24	39	5	2013.4
567	infectious disease	1	46	213	38	20.200.526
575	infectious diseases	3	25	43	8	2018.25
577	influenza	1	25	41	5	2020
610	internet	3	33	64	7	20.174.286

647	learning systems	1	26	52	7	20.202.857
668	machine learning	1	36	97	17	2021
702	medical information	4	41	76	6	20.198.333
716	mental health	4	19	25	5	2021
776	natural language processing	1	40	109	14	20.209.286
851	pandemic	4	45	190	24	20.212.917
874	perception	2	22	38	5	2017.8
899	pneumonia, viral	4	25	58	5	2020
933	prevention and control	2	29	67	7	20.202.857
940	procedures	3	26	34	5	2017.2
953	psychology	2	43	111	11	20.190.909
957	public health	1	51	191	27	20.194.815
967	public opinion	3	33	75	12	20.180.833
971	public policy	1	20	27	6	20.156.667
1047	sars-cov-2	2	31	98	9	20.214.444
1065	sentiment analysis	1	51	301	54	20.209.444
1097	social media	1	53	305	38	20.204.211
1104	social media platforms	1	19	41	6	2020.5
1109	social networking (online)	1	38	149	24	20.197.917
1188	text mining	1	24	34	7	20.208.571
1244	twitter	1	41	113	16	20.196.875
1255	united states	3	30	72	9	20.147.778
1273	vaccination	2	41	143	17	20.192.941
1277	vaccine	2	32	84	9	2019
1281	vaccine hesitancy	2	31	89	9	20.214.444
1285	vaccines	2	38	87	11	20.179.091
1303	virus pneumonia	4	25	58	5	2020
1307	viruses	1	20	54	9	20.205.556
