# Supplementary material for: Sentiment analysis of epidemiological surveillance reports on COVID-19 in Greece using machine learning models
Source: Front Public Health. 2023 Jul 18;11:1191730. doi: 10.3389/fpubh.2023.1191730 (PMC10392838; doi:10.3389/fpubh.2023.1191730)
Supplement: Supplementary file 3 [file Data_Sheet_3.pdf]

Supplementary Material

Sentiment Analysis of Epidemiological Surveillance Reports On COVID-19 In Greece Using Machine Learning Models

Christos Stefanis, Elpida Giorgi, Konstantinos Kalentzis, Athanasios Tselemponis, Evangelia Nena, Christina Tsigalou, Christos Kontogiorgis, Yiannis Kourkoutas, Aikaterini Chatzaki, Ioannis Dokas, Theodoros Constantinidis and Eugenia Bezirtzoglou*

*** Correspondence:** Eugenia Bezirtzoglou:empezirt@yahoo.gr

Supplement 3. References

Research title	Citation
A Proposed Framework for Developing User-Centred Mobile Healthcare Applications for the Biggest Annual Mass Gathering (Hajj) Post COVID-19	Islam, Nowman, Anwar, Aamir and Rehman, Ikram (2021). A proposed framework for developing user-centred mobile healthcare applications for the biggest annual mass gathering (Hajj) post COVID-19. In: *34th British Human Computer Interaction Conference,* 19-21 Jul 2021, London, UK.
A Social Media Time-Series Data Analytics Approach for Digital Epidemiology	M. A. Parwez, M. Abulaish and J. Jahiruddin (2020). A Social Media Time-Series Data Analytics Approach for Digital Epidemiology," *2020 IEEE/WIC/ACM International Joint Conference on Web Intelligence and Intelligent Agent Technology (WI-IAT)*, Melbourne, Australia, 2020, pp. 852-859, doi: 10.1109/WIIAT50758.2020.00131.
Analyzing Twitter Data to Evaluate People's Attitudes towards Public Health Policies and Events in the Era of COVID-19	Tsai, M. H., & Wang, Y. (2021). Analyzing Twitter Data to Evaluate People's Attitudes towards Public Health Policies and Events in the Era of COVID-19. *International journal of environmental research and public health*, *18*(12), 6272. https://doi.org/10.3390/ijerph18126272.
Assessing Vaccination Sentiments with Online Social Media: Implications for Infectious Disease Dynamics and Control	Salathé, M., & Khandelwal, S. (2011). Assessing vaccination sentiments with online social media: implications for infectious disease dynamics and control. *PLoS computational biology*, *7*(10), e1002199. https://doi.org/10.1371/journal.pcbi.1002199.
CollaborativeHealth: Smart Technologies to Surveil Outbreaks of Infectious Diseases Through Direct and Indirect Citizen Participation	Apolinario-Arzube, Oscar & García-Díaz, José & Pinto, Sheila & Luna Aveiga, Harry & Medina-Moreira, José & Gómez Berbis, Juan & Valencia-García, Rafael & Estrade-Cabrera, José. (2020). CollaborativeHealth: Smart Technologies to Surveil Outbreaks of Infectious Diseases Through Direct and Indirect Citizen Participation. 10.1007/978-3-030-51974-2_15.
DiseaSE: A biomedical text analytics system for disease symptom extraction and characterization	Abulaish, M., Parwez, M. A., & Jahiruddin (2019). DiseaSE: A biomedical text analytics system for disease symptom extraction and characterization. *Journal of biomedical informatics*, *100*, 103324. https://doi.org/10.1016/j.jbi.2019.103324.
Effective surveillance and predictive mapping of mosquito-borne diseases using social media	Jain, V.K. and Kumar, S. (2018). Effective surveillance and predictive mapping of mosquito-borne diseases using social media. Journal of Computational Science, *25, pp. 406–415.* doi:10.1016/j.jocs.2017.07.003.
Evaluation of IBM Watson Natural Language Processing Service to predict influenza-like illness outbreaks from Twitter data	Kanita Karađuzović-Hadžiabdić, Rialda Spahić, Emin Tahirović (2022). Evaluation of IBM Watson Natural Language Processing Service to predict influenza-like illness outbreaks from Twitter data. Periodicals of Engineering and Natural Sciences Original Research,

	10, 1, January 2022, pp.122-137.
Fine-tuned Sentiment Analysis of COVID-19 Vaccine–Related Social Media Data: Comparative Study	Melton, C. A., White, B. M., Davis, R. L., Bednarczyk, R. A., & Shaban-Nejad, A. (2022). Fine-tuned Sentiment Analysis of COVID-19 Vaccine-Related Social Media Data: Comparative Study. Journal of medical Internet research, *24*(10), e40408. https://doi.org/10.2196/40408.
Identification and characterization of tweets related to the 2015 Indiana HIV outbreak: A retrospective infoveillance study	Cai, M., Shah, N., Li, J., Chen, W. H., Cuomo, R. E., Obradovich, N., & Mackey, T. K. (2020). Identification and characterization of tweets related to the 2015 Indiana HIV outbreak: A retrospective infoveillance study. *PloS one*, *15*(8), e0235150. https://doi.org/10.1371/journal.pone.0235150.
Infectious or Recovered? Optimizing the Infectious Disease Detection Process for Epidemic Control and Prevention Based on Social Media	Shan, S., Yan, Q., & Wei, Y. (2020). Infectious or Recovered? Optimizing the Infectious Disease Detection Process for Epidemic Control and Prevention Based on Social Media. International journal of environmental research and public health, *17*(18), 6853. https://doi.org/10.3390/ijerph17186853.
Influenza-like Illness Detection from Arabic Facebook Posts Based on Sentiment Analysis and 1D Convolutional Neural Network	Boulesnane, Abdennour, Souham Meshoul, and Khaoula Aouissi (2022). Influenza-like Illness Detection from Arabic Facebook Posts Based on Sentiment Analysis and 1D Convolutional Neural Network. Mathematics *10, no. 21: 4089*. https://doi.org/10.3390/math10214089.
Information-Seeking Patterns During the COVID-19 Pandemic Across the United States: Longitudinal Analysis of Google Trends Data	Mangono, T., Smittenaar, P., Caplan, Y., Huang, V. S., Sutermaster, S., Kemp, H., & Sgaier, S. K. (2021). Information-Seeking Patterns During the COVID-19 Pandemic Across the United States: Longitudinal Analysis of Google Trends Data. Journal of medical Internet research, *23*(5), e22933. https://doi.org/10.2196/22933.
Leveraging social networking sites for disease surveillance and public sensing: the case of the 2013 avian influenza A(H7N9) outbreak in China	Zhang, E. X., Yang, Y., Di Shang, R., Simons, J. J., Quek, B. K., Yin, X. F., See, W., Oh, O. S., Nandar, K. S., Ling, V. R., Chan, P. P., Wang, Z., Goh, R. S., James, L., & Tey, J. S. (2015). Leveraging social networking sites for disease surveillance and public sensing: the case of the 2013 avian influenza A(H7N9) outbreak in China. Western Pacific surveillance and response journal : WPSAR, 6(2), 66–72. https://doi.org/10.5365/WPSAR.2015.6.1.013
Main uses of Instagram in oral health research–A scoping review	Leandro Machado Oliveira, Kimberly da Silva Pilecco, Daniel Fagundes de Souza, Cícero Anghinoni de Oliveira, Fabrício Batistin Zanatta, Main uses of Instagram in oral health research–A scoping review (2022). Health Policy and Technology, 11, 1, 100605, ISSN 2211-8837, https://doi.org/10.1016/j.hlpt.2022.100605.
Mining topic and sentiment dynamics in physician rating websites during the early wave of the COVID-19 pandemic: Machine learning approach	Shah, A. M., Yan, X., Qayyum, A., Naqvi, R. A., & Shah, S. J. (2021). Mining topic and sentiment dynamics in physician rating websites during the early wave of the COVID-19 pandemic: Machine learning approach. International Journal of Medical Informatics, *149*, *[104434]*. https://doi.org/10.1016/j.ijmedinf.2021.104434
Mining Twitter Data For Influenza Detection and Surveillance	Kenny Byrd, Alisher Mansurov, and Olga Baysal. (2016). Mining Twitter data for influenza detection and surveillance. In Proceedings of the International Workshop on Software Engineering in Healthcare Systems (SEHS '16). Association for Computing Machinery, New York, NY, USA, 43–49. https://doi.org/10.1145/2897683.2897693.
Multi-perspectives systematic review on the applications of sentiment analysis for vaccine hesitancy	Alamoodi, A. H., Zaidan, B. B., Al-Masawa, M., Taresh, S. M., Noman, S., Ahmaro, I. Y. Y., Garfan, S., Chen, J., Ahmed, M. A., Zaidan, A. A., Albahri, O. S., Aickelin, U., Thamir, N. N., Fadhil, J. A., & Salahaldin, A. (2021). Multi-perspectives systematic review on the applications of sentiment analysis for vaccine hesitancy. Computers in biology and medicine, *139*, *104957*. https://doi.org/10.1016/j.compbiomed.2021.104957
Prediction of Influenza-like Illness	Malik, M., Naaz, S. (2021). Prediction of Influenza-like Illness from

from Twitter Data and Its Comparison with Integrated Disease Surveillance	Twitter Data and Its Comparison with Integrated Disease Surveillance Program Data. In: Pandian, A., Fernando, X., Islam, S.M.S. (eds) Computer Networks, Big Data and IoT. Lecture Notes on Data Engineering and Communications Technologies, vol 66. Springer, Singapore. https://doi.org/10.1007/978-981-16-0965-7_31.
PREDOSE: A semantic web platform for drug abuse epidemiology using social media	Cameron, D., Smith, G. A., Daniulaityte, R., Sheth, A. P., Dave, D., Chen, L., Anand, G., Carlson, R., Watkins, K. Z., & Falck, R. (2013). PREDOSE: a semantic web platform for drug abuse epidemiology using social media. Journal of biomedical informatics, *46*(6), 985–997. https://doi.org/10.1016/j.jbi.2013.07.007.
Preliminary Flu Outbreak Prediction Using Twitter Posts Classification and Linear Regression With Historical Centers for Disease Control and Prevention Reports: Prediction Framework Study	Alessa, A., & Faezipour, M. (2019). Flu Outbreak Prediction Using Twitter Posts Classification and Linear Regression With Historical Centers for Disease Control and Prevention Reports: Prediction Framework Study. *JMIR public health and surveillance*, *5*(2), e12383. https://doi.org/10.2196/12383.
Real-Time Infoveillance of Moroccan Social Media Users' Sentiments towards the COVID-19 Pandemic and Its Management	Ghanem, A., Asaad, C., Hafidi, H., Moukafih, Y., Guermah, B., Sbihi, N., Zakroum, M., Ghogho, M., Dairi, M., Cherqaoui, M., & Baina, K. (2021). Real-Time Infoveillance of Moroccan Social Media Users' Sentiments towards the COVID-19 Pandemic and Its Management. International journal of environmental research and public health, *18(22), 12172*. https://doi.org/10.3390/ijerph182212172.
Rise and fall of the global conversation and shifting sentiments during the COVID-19 pandemic	Zhang, X., Yang, Q., Albaradei, S. *et al.* Rise and fall of the global conversation and shifting sentiments during the COVID-19 pandemic. *Humanit Soc Sci Commun* **8**, 120 (2021). https://doi.org/10.1057/s41599-021-00798-7.
Sentiment Analysis as a Service: A social media based sentiment analysis framework	K. Ali, H. Dong, A. Bouguettaya, A. Erradi and R. Hadjidj, "Sentiment Analysis as a Service: A Social Media Based Sentiment Analysis Framework," *2017 IEEE International Conference on Web Services (ICWS)*, Honolulu, HI, USA, 2017, pp. 660-667, doi: 10.1109/ICWS.2017.79.
Sentiment analysis of tweets on alopecia areata, hidradenitis, suppurativa, and psoriasis: Revealing the patient experience	Lee, I. T., Juang, S. E., Chen, S. T., Ko, C., & Ma, K. S. (2022). Sentiment analysis of tweets on alopecia areata, hidradenitis suppurativa, and psoriasis: Revealing the patient experience. *Frontiers in medicine*, *9*, 996378. https://doi.org/10.3389/fmed.2022.996378.
Sentiment-Based Spatiotemporal Prediction Framework for Pandemic Outbreaks Awareness Using Social Networks Data Classification	Xie, Tianyi, Yaorong Ge, Qian Xu, and Shi Chen. (2023). Public Awareness and Sentiment Analysis of COVID-Related Discussions Using BERT-Based Infoveillance. *AI* 4, no. 1: 333-347. https://doi.org/10.3390/ai4010016.
Surveilling COVID-19 Emotional Contagion on Twitter by Sentiment Analysis	Crocamo, C., Viviani, M., Famiglini, L., Bartoli, F., Pasi, G., & Carrà, G. (2021). Surveilling COVID-19 Emotional Contagion on Twitter by Sentiment Analysis. *European psychiatry : the journal of the Association of European Psychiatrists*, *64*(1), e17. https://doi.org/10.1192/j.eurpsy.2021.3.
Text Classification of Flu-related Tweets Using FastText with Sentiment and Keyword Features	A. Alessa, M. Faezipour and Z. Alhassan, "Text Classification of Flu-Related Tweets Using FastText with Sentiment and Keyword Features," 2018 IEEE International Conference on Healthcare Informatics (ICHI), New York, NY, USA, 2018, pp. 366-367, doi: 10.1109/ICHI.2018.00058.
Text mining in mosquito-borne disease: A systematic review	Ong, S. Q., Pauzi, M. B. M., & Gan, K. H. (2022). Text mining in mosquito-borne disease: A systematic review. *Acta tropica*, *231*, 106447. https://doi.org/10.1016/j.actatropica.2022.106447
Top Concerns of Tweeters During the COVID-19 Pandemic: Infoveillance Study	Abd-Alrazaq A, Alhuwail D, Househ M, Hamdi M, Shah Z. (2020). Top Concerns of Tweeters During the COVID-19 Pandemic: Infoveillance Study

	J Med Internet Res, 22(4):e19016<br>URL: https://www.jmir.org/2020/4/e19016<br>DOI: 10.2196/19016.
Topics, Trends, and Sentiments of Tweets About the COVID-19 Pandemic: Temporal Infoveillance Study	Chandrasekaran, R., Mehta, V., Valkunde, T., & Moustakas, E. (2020). Topics, Trends, and Sentiments of Tweets About the COVID-19 Pandemic: Temporal Infoveillance Study. *Journal of medical Internet research*, *22*(10), e22624. https://doi.org/10.2196/22624.
Twitter Catches The Flu: Detecting Influenza Epidemics using Twitter	Eiji Aramaki, Sachiko Maskawa, and Mizuki Morita. 2011. Twitter Catches The Flu: Detecting Influenza Epidemics using Twitter. In Proceedings of the 2011 Conference on Empirical Methods in Natural Language Processing, pages 1568–1576, Edinburgh, Scotland, UK. Association for Computational Linguistics.
Twitter sentiment classification for measuring public health concerns	Ji, X., Chun, S. A., Wei, Z., & Geller, J. (2015). Twitter sentiment classification for measuring public health concerns. Social network analysis and mining, 5(1), 13. https://doi.org/10.1007/s13278-015-0253-5.
Using a mixed methods approach to identify public perception of vaping risks and overall health outcomes on Twitter during the 2019 EVALI outbreak	Kasson, E., Singh, A. K., Huang, M., Wu, D., & Cavazos-Rehg, P. (2021). Using a mixed methods approach to identify public perception of vaping risks and overall health outcomes on Twitter during the 2019 EVALI outbreak. International journal of medical informatics, 155, 104574. https://doi.org/10.1016/j.ijmedinf.2021.104574.
Using electronic health records and Internet search information or accurate influenza forecasting	Yang, S., Santillana, M., Brownstein, J. S., Gray, J., Richardson, S., & Kou, S. C. (2017). Using electronic health records and Internet search information for accurate influenza forecasting. *BMC infectious diseases*, *17*(1), 332. https://doi.org/10.1186/s12879-017-2424-7.
